# Complete Genome Sequence of the Lignocellulose-Degrading Actinomycete Streptomyces albus CAS922

**DOI:** 10.1128/MRA.00227-20

**Published:** 2020-05-21

**Authors:** Anna Tippelt, Markus Nett, M. Soledad Vela Gurovic

**Affiliations:** a Department of Biochemical and Chemical Engineering, TU Dortmund University, Dortmund, Germany; b Center for Renewable Natural Resources of the Semi-Arid Zone, National Scientific and Technical Research Council, Bahía Blanca, Argentina; c Department of Biology, Biochemistry, and Pharmacy, Universidad Nacional del Sur, Bahía Blanca, Argentina; University of Arizona

## Abstract

Streptomyces albus CAS922 was isolated from sunflower seed hulls. Its fully sequenced genome harbors a multitude of genes for carbohydrate-active enzymes, which likely facilitate growth on lignocellulosic biomass. Furthermore, the presence of 27 predicted biosynthetic gene clusters indicates a significant potential for the production of bioactive secondary metabolites.

## ANNOUNCEMENT

Actinomycete bacteria are important contributors to the natural degradation of lignocellulosic biomass (1, 2). An illustrative example is Streptomyces albus CAS922, which was isolated from sunflower seed hulls discarded by the sunflower oil industry (Cargill S.A.C.I., Bahía Blanca, Argentina) by using ISP-2 agar plates (3) and has been found to grow on diverse lignocellulosic substrates. The strain produces an earthy odor and shows typical streptomycete morphology, including aerial mycelia and substrate hyphae penetrating the agar. Since many actinomycetes are versatile producers of clinically used antibiotics (4, 5), S. albus CAS922 might possess the potential to convert lignocellulosic biomass into value-added products. To obtain data to test this hypothesis, its genome was sequenced.

For DNA isolation, a glycerol stock of S. albus CAS922 was incubated in ISP-2 broth at 30°C and 250 rpm for 4 days. Genomic DNA was then isolated according to a protocol adapted from a report by Neumann et al. with minor modifications (6). Briefly, cells were harvested from liquid ISP-2 cultures and ground in liquid nitrogen in a mortar. The sample was incubated with lysozyme for 2 h at 300 rpm and proteinase K for 3 h at 300 rpm and was extracted with a mixture of phenol, chloroform, and isoamyl alcohol (25:24:1) followed by RNase treatment. Genome sequencing was executed by CD Genomics (New York, NY, USA) using the Oxford Nanopore sequencing platform. The SQK-LSK109 kit was used for library preparation following the 1D genomic DNA by ligation protocol (7). Adapters were filtered with the software Porechop v0.2.3. Low-quality and short-fragment (<2,000-bp) reads were removed with the MinKNOW software package to obtain a total of 1,543,556,228 bp of clean data from 260,187 clean reads with an *N*_50_ value of 7,942 bp. The clean reads were assembled using Canu v1.5 software (8), followed by a subsequent data-polishing step with Pilon software (9) to increase accuracy. This resulted in a linear chromosome of 8.06 Mb with 191-fold genome coverage and a G+C content of 72.59%. The Prokaryotic Genome Annotation Pipeline (PGAP) (10) predicted 6,776 protein-coding genes, 80 RNAs, 59 tRNAs, 3 noncoding RNAs, and 312 pseudogenes. A repetitive sequence content of 2.45% was predicted by RepeatMasker (11). Assignment of the strain to the species S. albus was verified based on alignments with the nonredundant database (12). Default parameters were used for all software.

A total of 232 carbohydrate-active enzymes were predicted using the CAZy database (13) and dbCAN2 (14). Three of these proteins belong to the AA10 family, recently recognized as copper-dependent lytic polysaccharide monooxygenases involved in the lysis of chitin, cellulose, and xylan (15).

An antiSMASH analysis (16) revealed 27 putative biosynthetic gene clusters. According to this prediction, S. albus CAS922 produces the siderophore desferrioxamine E (17), the compatible solute ectoine (18), and the terpenes cyslabdan (19) and geosmin (20). Moreover, the actinomycete harbors the genes for biosynthesis of the antibiotics xantholipin (21) and pseudouridimycin (22). Overall, S. albus CAS922 possesses a high potential for the utilization of lignocellulose and the biosynthesis of secondary metabolites.

### Data availability.

This whole-genome project has been deposited in GenBank with the accession number CP048875. The raw data are available under accession number SRR11069776.

## References

[1] Antunes PL, Martins FL, Pereira RV, Thomas AM, Barbosa D, Lemos LN, Machado Silva GM, Silva Moura LM, Condomitti Epamino GW, Digiampietri LA, Lombardi KC, Locosque Ramos P, Bento Quaggio R, Franco de Oliveira JC, Castiglioni Pascon R, da Cruz JB, da Silva AM, Setubal JC 2016 Microbial community structure and dynamics in thermophilic composting viewed through metagenomics and metatranscriptomics. Sci Rep 6:38915. doi:10.1038/srep38915. 27941956 PMC5150989

[2] Větrovský T, Steffen KT, Baldrian P 2014 Potential of cometabolic transformation of polysaccharides and lignin in lignocellulose by soil actinobacteria. PLoS One 9:e89108. doi:10.1371/journal.pone.0089108. 24551229 PMC3923840

[3] Shirling EB, Gottlieb D 1966 Methods for characterization of *Streptomyces* species. Int J Syst Bact 16:313–340. doi:10.1099/00207713-16-3-313.

[4] van der Meij A, Worsley SF, Hutchings MI, van Wezel GP 2017 Chemical ecology of antibiotic production by actinomycetes. FEMS Microbiol Rev 41:392–416. doi:10.1093/femsre/fux005. 28521336

[5] Nett M, Ikeda H, Moore BS 2009 Genomic basis for natural product biosynthetic diversity in the actinomycetes. Nat Prod Rep 26:1362–1384. doi:10.1039/b817069j. 19844637 PMC3063060

[6] Neumann B, Pospiech A, Schairer HU 1992 Rapid isolation of genomic DNA from Gram-negative bacteria. Trends Genet 8:332–333. doi:10.1016/0168-9525(92)90269-A. 1475843

[7] Oxford Nanopore Technologies. 2020 Genomic DNA by ligation (SQK-LSK109). Oxford Nanopore Technologies, Oxford, United Kingdom https://store.nanoporetech.com/us/media/wysiwyg/pdfs/SQK-LSK109/Genomic_DNA_by_Ligation_SQK-LSK109_-minion.pdf.

[8] Koren S, Walenz BP, Berlin K, Miller JR, Bergman NH, Phillippy AM 2017 Canu: scalable and accurate long-read assembly via adaptive *k*-mer weighting and repeat separation. Genome Res 27:722–736. doi:10.1101/gr.215087.116. 28298431 PMC5411767

[9] Walker BJ, Abeel T, Shea T, Priest M, Abouelliel A, Sakthikumar S, Cuomo CA, Zeng Q, Wortman J, Young SK, Earl AM 2014 Pilon: an integrated tool for comprehensive microbial variant detection and genome assembly improvement. PLoS One 9:e112963. doi:10.1371/journal.pone.0112963. 25409509 PMC4237348

[10] Tatusova T, DiCuccio M, Badretdin A, Chetvernin V, Nawrocki EP, Zaslavsky L, Lomsadze A, Pruitt KD, Borodovsky M, Ostell J 2016 NCBI Prokaryotic Genome Annotation Pipeline. Nucleic Acids Res 44:6614–6624. doi:10.1093/nar/gkw569. 27342282 PMC5001611

[11] Tarailo‐Graovac M, Chen N 2009 Using RepeatMasker to identify repetitive elements in genomic sequences. Curr Protoc Bioinformatics 4.10.1–4.10.14. doi:10.1002/0471250953.bi0410s25. 19274634

[12] Deng YY, Li JQ, Wu SF, Zhu YP, Chen YW, He FC 2006 Integrated nr database in protein annotation system and its localization. Comput Eng 32:71–74.

[13] Cantarel BL, Coutinho PM, Rancurel C, Bernard T, Lombard V, Henrissat B 2009 The Carbohydrate-Active EnZymes database (CAZy): an expert resource for glycogenomics. Nucleic Acids Res 37:D233–D238. doi:10.1093/nar/gkn663. 18838391 PMC2686590

[14] Zhang H, Yohe T, Huang L, Entwistle S, Wu P, Yang Z, Busk PK, Xu Y, Yin Y 2018 dbCAN2: a meta server for automated carbohydrate-active enzyme annotation. Nucleic Acids Res 46:W95–W101. doi:10.1093/nar/gky418. 29771380 PMC6031026

[15] Corrêa TLR, Júnior AT, Wolf LD, Buckeridge MS, Dos Santos LV, Murakami MT 2019 An actinobacteria lytic polysaccharide monooxygenase acts on both cellulose and xylan to boost biomass saccharification. Biotechnol Biofuels 12:117. doi:10.1186/s13068-019-1449-0. 31168322 PMC6509861

[16] Blin K, Shaw S, Steinke K, Villebro R, Ziemert N, Lee SY, Medema MH, Weber T 2019 antiSMASH 5.0: updates to the secondary metabolite genome mining pipeline. Nucleic Acids Res 47:W81–W87. doi:10.1093/nar/gkz310. 31032519 PMC6602434

[17] Barona-Gómez F, Wong U, Giannakopulos AE, Derrick PJ, Challis GL 2004 Identification of a cluster of genes that directs desferrioxamine biosynthesis in *Streptomyces coelicolor* M145. J Am Chem Soc 126:16282–16283. doi:10.1021/ja045774k. 15600304

[18] Louis P, Galinski EA 1997 Characterization of genes for the biosynthesis of the compatible solute ectoine from *Marinococcus halophilus* and osmoregulated expression in *Escherichia coli*. Microbiology 143:1141–1149. doi:10.1099/00221287-143-4-1141. 9141677

[19] Ikeda H, Shin-Ya K, Nagamitsu T, Tomoda H 2016 Biosynthesis of mercapturic acid derivative of the labdane-type diterpene, cyslabdan that potentiates imipenem activity against methicillin-resistant *Staphylococcus aureus*: cyslabdan is generated by mycothiol-mediated xenobiotic detoxification. J Ind Microbiol Biotechnol 43:325–342. doi:10.1007/s10295-015-1694-6. 26507838

[20] Jiang J, He X, Cane DE 2007 Biosynthesis of the earthy odorant geosmin by a bifunctional *Streptomyces coelicolor* enzyme. Nat Chem Biol 3:711–715. doi:10.1038/nchembio.2007.29. 17873868 PMC3013058

[21] Zhang W, Wang L, Kong L, Wang T, Chu Y, Deng Z, You D 2012 Unveiling the post-PKS redox tailoring steps in biosynthesis of the type II polyketide antitumor antibiotic xantholipin. Chem Biol 19:422–432. doi:10.1016/j.chembiol.2012.01.016. 22444597

[22] Sosio M, Gaspari E, Iorio M, Pessina S, Medema MH, Bernasconi A, Simone M, Maffioli SI, Ebright RH, Donadio S 2018 Analysis of the pseudouridimycin biosynthetic pathway provides insight into the formation of C-nucleoside antibiotics. Cell Chem Biol 25:540–549. doi:10.1016/j.chembiol.2018.02.008. 29551347 PMC5959762

